# Building capacity for evidence informed decision making in public health: a case study of organizational change

**DOI:** 10.1186/1471-2458-12-137

**Published:** 2012-02-20

**Authors:** Leslea Peirson, Donna Ciliska, Maureen Dobbins, David Mowat

**Affiliations:** 1 McMaster University School of Nursing, Hamilton, ON, Canada; 2 National Collaborating Centre for Methods and Tools, McMaster University, 1685 Main St., W., Suite 302, Hamilton, ON, Canada L8S 1G5; 3 Peel Public Health Department, Brampton, ON, Canada

## Abstract

**Background:**

Core competencies for public health in Canada require proficiency in evidence informed decision making (EIDM). However, decision makers often lack access to information, many workers lack knowledge and skills to conduct systematic literature reviews, and public health settings typically lack infrastructure to support EIDM activities. This research was conducted to explore and describe critical factors and dynamics in the early implementation of one public health unit's strategic initiative to develop capacity to make EIDM standard practice.

**Methods:**

This qualitative case study was conducted in one public health unit in Ontario, Canada between 2008 and 2010. In-depth information was gathered from two sets of semi-structured interviews and focus groups (n = 27) with 70 members of the health unit, and through a review of 137 documents. Thematic analysis was used to code the key informant and document data.

**Results:**

The critical factors and dynamics for building EIDM capacity at an organizational level included: clear vision and strong leadership, workforce and skills development, ability to access research (library services), fiscal investments, acquisition and development of technological resources, a knowledge management strategy, effective communication, a receptive organizational culture, and a focus on change management.

**Conclusion:**

With leadership, planning, commitment and substantial investments, a public health department has made significant progress, within the first two years of a 10-year initiative, towards achieving its goal of becoming an evidence informed decision making organization.

## Background

In recent years, public health systems have confronted outbreaks of infectious diseases, water and food-borne contamination/illnesses, parasitic infestations and other exigent epidemics. Prompt and effective decisions and actions are needed to address these crises and promote the well-being of individuals, families and communities. However, in urgent and routine situations decision makers often lack access to important information, many workers do not have the knowledge and skills to conduct systematic reviews of the research literature, and organizations typically lack infrastructure to support these activities [1-7]. In public health, the term "evidence informed decision making" (EIDM) refers to intentional and systematic processes of bringing the best available scientific evidence on specific questions together with other relevant information to help weigh options and inform decisions that will affect priorities, policies, programs and practices [8-11].

Recent changes indicate that developing capacity for EIDM has become a priority for public health in Canada. For instance, in 2005 six National Collaborating Centres for Public Health were established to help translate scientific evidence so it can be used by policy makers and practitioners to respond effectively and efficiently to chronic and infectious diseases, injuries, environmental risks, and health and social disparities [12]. The 2008 national core competency statements for public health asserted practitioners must have the skills, knowledge and attitudes necessary to engage in EIDM [13]. Similarly, the most recent Ontario Public Health Standards emphasized evidence informed practice as a foundation for all mandated programs and services in the province [14]. A variety of training modules and workshops have also been introduced to support EIDM skills development in the public health workforce [e.g., [9,15-18]]. The public health sector is clearly moving forward on its EIDM objectives; yet local public health units continue to experience implementation difficulties [4,19,20]. Attention must be given to infrastructure development and creating contexts that promote EIDM as routine practice [1,21].

Some health care settings have taken up this challenge. For example, after recognizing a disconnect between new knowledge, research and innovation and its existing operations, a Regional Health Authority in Eastern Canada initiated strategic efforts to foster an organizational culture that supports research and learning, invested in tools, training and mentoring for EIDM, and created interdisciplinary working groups to promote the integration and application of research and research evidence in decision making and practice [22]. To operationalize its vision of becoming "a phenomenal knowledge and care exchange company," senior leaders of a large homecare organization in Ontario advanced a multi-stage process of systems change that involved building a culture of critical inquiry, developing staff skills and piloting tools for EIDM, improving capacity to access evidence, and introducing a new staff unit to focus on the organization's knowledge management and translation needs [23]. By reallocating existing salary dollars, an Alberta rehabilitation service organization created a Time Grants program that provides individual employees or teams of staff with substantial, dedicated and protected time, along with mentoring and other supports, to work on projects that evaluate, use and/or produce research evidence to addresses issues relevant to the organization's practice [24]. No formal evaluations of the impacts of these initiatives have been conducted and/or published, however early reports from these organizations indicate promising results in terms facilitating important changes in clinical and management practices, increasing staff skills and confidence, and generating widespread stakeholder buy-in for EIDM approaches. An integrated knowledge translation [25] and intervention research project focused on building organizational capacity for EIDM is currently underway in three contextually diverse public health units in Ontario [26]. The results of this multi-site case study will provide much needed comparisons of the impacts and outcomes of EIDM and the contextual factors that facilitate or challenge EIDM activities which should increase the transferability of the findings for other health care settings.

The intent of this research was to explore and inform the early implementation of one public health unit's long-term strategic initiative to build organization and staff capacity for EIDM. The main focus of this paper is to present the findings related to critical factors and dynamics involved in the organization's transformation to advance EIDM objectives.

## Methods

### Setting and context

Peel Public Health (PPH) is the second largest health unit in Ontario and one of the largest in Canada. The 800 staff serve 1.3 million residents of Peel Region, a mostly urban area west of Toronto. In 2007, shortly after a new Medical Officer of Health (MOH) was hired, PPH initiated a process to develop a comprehensive strategic plan to guide organizational priorities and activities over the next decade. After 18 months of extensive planning, consultations and vetting processes, the Strategic Plan for 2009-2019 was officially launched [27]. Along with several program priorities, the Plan emphasized five infrastructure priorities: EIDM, Workforce Development, Performance Management, Communication and Ethno-Cultural Diversity. With respect to EIDM, the Plan affirmed a commitment to develop capacity to ensure relevant, high quality research evidence is considered a key input for decision making.

### Design

The research involved a longitudinal, single-case study with an organization as the unit of analysis [28-30]. Case-study design was appropriate to develop an in-depth understanding of the organization's approach for incorporating research evidence into decision making processes, to explore organization and staff capacity for EIDM, to examine changes in capacity over time, and to identify the factors and dynamics that influenced both capacity development and change. "Within-method triangulation" [31] was used to gather in-depth information at multiple points in time from multiple sources, including: two rounds of interviews and focus groups and two rounds of documentary review. The research also used an integrated knowledge translation approach which means members of the organization who would use the study's findings to inform on-going implementation of the EIDM initiative, including one of the authors of this paper (DM), were involved in designing the study, collaborating on the research questions and methods, validating and interpreting the findings, and disseminating the results [25]. The study was approved by the Hamilton Health Sciences/Faculty of Health Sciences (McMaster University) Research Ethics Board.

### Participants and recruitment

A mixed purposeful sampling strategy [28] was used to identify individuals responsible for the EIDM component of the Strategic Plan, as well as staff who conduct literature reviews and their supervisors. We chose these stakeholders since they were most intimately involved in and/or targeted by the EIDM initiative in its early stages. Concentrated efforts to inform and involve field-level personnel were planned for a later phase; as such we did not recruit these staff. The sample included the Medical Officer of Health (MOH), Associate Medical Officers of Health (AMOHs), Library Personnel, and Directors, Managers, Supervisors, and Specialists (e.g., Research and Policy Analysts, Project Specialists, Epidemiologists) from all divisions. A total of 70 key informants participated in this study; 58 in the first round of data collection, 42 in the second round, and 30 at both times. Tables 1, 2 and 3 show the distribution of participants across affiliations, roles and service divisions. All participants were recruited by the primary author who described the study and the efforts that would be taken to protect participants' privacy and anonymity and to ensure the confidentiality of the data.

**Table 1:** Key informant sample by affiliation

	1	2	3	4	5	6
**1**	56	27	40	11	29	67
**2**	1	1	0	0	0	1
**3**	1	0	2	1	1	2
**4**	58	28	42	12	30	70

Vertical Axis--Affiliations: 1--PPH employees; 2--Region of Peel (not PPH) employees; 3--External consultants; 4--Total number of participants in data collection period(s)

Horizontal Axis--Number of Participants in Data Collection Period(s): 1--Participated 2008; 2--Participated 2008 only; 3--Participated 2010; 4--Participated 2010 only; 5--Participated 2008 and 2010; 6--Total number of participants by affiliation

**Table 2:** Key informant sample by role

	1	2	3	4	5	6
**1**	23	11	17	5	12	28
**2**	12	7	10	5	5	17
**3**	14	11	5	2	3	16
**4**	4	1	3	0	3	4
**5**	3	0	4	1	3	4
**6**	2	1	3	2	1	4
**7**	1	0	2	1	1	2
**8**	58	28	42	12	30	70

Vertical Axis--Roles: 1--Specialists (Research and Policy Analysts, Health Promotion Officers, Project Specialists, Epidemiologists); 2--Managers; 3--Supervisors; 4--Directors; 5--Medical Officers of Health; 6--Library Personnel (Includes Librarian in 2008 and 2010; 2 Managers, 1 in 2008, 1 in 2010; 1 Consultant in 2010); 7--Consultants; 8--Total number of participants in data collection period(s)

Horizontal Axis--Number of Participants in Data Collection Period(s): 1--Participated 2008; 2--Participated 2008 only; 3--Participated 2010; 4--Participated 2010 only; 5--Participated 2008 and 2010; 6--Total number of participants by role (Total > 70: 2 people participated in 2008 and 2010 but occupied different roles and are counted in each category; 2 participants counted in both Manager and Library categories, 1 in 2008, 1 in 2010; 1 Consultant counted in 2010 Library category)

**Table 3:** Key informant sample by division

	1	2	3	4	5	6
**1**	6	0	10	4	6	10
**2**	21	15	9	3	6	24
**3**	14	7	7	0	7	14
**4**	6	1	7	1	6	8
**5**	7	5	6	4	2	11
**6**	2	1	2	1	1	3
**7**	58	28	42	12	30	70

Vertical Axis--Divisions: 1--Office of the MOH (Includes MOH and AMOHs, 2 Specialists, Manager of Research & Education, Epidemiologists); 2--Chronic Diseases & Injury Prevention; 3--Communicable Diseases; 4--Environmental Health; 5--Family Health; 6--Library Services (Includes Librarian in 2008 and 2010; 2 Managers, 1 in 2008, 1 in 2010); 7--Total number of participants in data collection period(s)

Horizontal Axis--Number of Participants in Data Collection Period(s): 1--Participated 2008; 2--Participated 2008 only; 3--Participated 2010; 4--Participated 2010 only; 5--Participated 2008 and 2010; 6--Total number of participants by division (Total = 70 but absolute number = 68: excludes consultants; the Region but non-PPH employee included under Library; one person triple-counted as Family Health, Office of the MOH and Library)

### Interviews and focus groups

Six interviews and 21 focus groups were conducted between September 2008 and February 2010. The first round of data collection (four interviews, nine focus groups) was undertaken in fall 2008, prior to the launch of the Strategic Plan and intensive efforts to advance the EIDM objectives. The purpose of these sessions was to: assess participants' understanding of EIDM, gauge awareness of the organization's plans regarding EIDM, construct a baseline picture of how evidence was typically brought into decision making processes across the organization, and identify facilitators and barriers of these activities. A second set of interviews (n = 2) and focus groups (n = 12) took place in winter 2010 to capture participants' thoughts on if/how the organization, its workforce, and they themselves had changed over the last 18 months with respect to EIDM.

Except for two telephone interviews, all sessions were conducted face-to-face in private rooms at PPH; most lasted one hour, although the duration ranged from 50 minutes to two hours. All sessions were conducted by the primary author (LP) who provided an overview of the study and the interview guide and reviewed ethical and procedural aspects for voluntary participation, recording, transcription and data validation. Participants were given the opportunity to ask questions about the research and each person completed a consent form.

The semi-structured sessions were guided by a series of open-ended questions designed to: elicit perceptions of and experiences with the EIDM initiative; identify methods, tools and resources used and needed to carry out reviews of the literature and other EIDM tasks; explore challenges and facilitators of EIDM activities; and prompt thoughts about the future of the EIDM initiative (see Additional file 1). The questions in the interview guide followed the stages in the evidence informed public health process as promoted by the National Collaborating Centre for Methods and Tools [32]. The 2008 sessions focused primarily on how staff engaged in the various steps of bringing evidence into decision making processes (e.g., accessing, appraising, applying) while the 2010 discussions focused more on the recent activities and dynamics involved in organizational change to promote EIDM.

### Documents

The purpose of the document review was to examine if/how the concept and approaches of EIDM were incorporated in the organization's written products, and to discern changes in the presence of evidence and EIDM over time. In 2008 and 2010, representatives were asked to provide a sample of recent documents, including: (1) business, operational and program plans; literature reviews; proposals for new programs, changes to existing programs, or to end programs; (2) job descriptions and performance appraisal criteria for the Specialist roles; (3) organization-level documents (e.g., organizational chart, strategic plan); and (4) documents produced for/by the EIDM initiative (e.g., assessments of the organization's EIDM capacity; minutes of working group meetings, tools for conducting literature reviews). In total, 137 documents were submitted; 67 in the first round and 70 in the second round.

### Data analysis

Each participant was provided with a transcript of their session and was asked to validate the accuracy, clarity and completeness of the data and to mark passages they did not want quoted directly. NVivo8 (QSR International), a qualitative analysis software, was used to organize, manage and code the validated key informant data. Key sources on systems change and learning organizations [e.g., [33-38]] and models of evidence informed practice [e.g., [25,32,39,40]] were used to develop a preliminary code list. Coding and interpretation were also inductive, allowing themes and sub-themes to emerge directly from the data [28]. In line with the questions in the interview guide, the first author analyzed the 2008 transcripts to draw out themes relating to: the methods, tools, skills and resources used or needed to bring evidence into decision making; the facilitators and barriers of EIDM activities; the EIDM strategic initiative; and the organizational context of the change. The resulting code list was discussed with two other authors (DC, MD) before being used to analyze the 2010 transcripts.

For the document analysis, the first author reviewed each source to extract relevant information in summary or in vivo form. Questions guiding the review included: How/Is evidence or EIDM included? What kinds/levels of evidence are used? How is evidence reported? What sources are consulted? Is there any critical appraisal of the evidence? How is evidence weighted against other factors in decision making? Notes from the submitted documents were entered into data extraction tables created with MSWord. The completed tables were used to conduct cross-document thematic analyses, the results of which were discussed with two other authors (DC, MD) before finalizing.

During the course of the research organizational efforts were concentrated on building infrastructure and preparing the context for change. As data collection for this study concluded the organization was shifting into a new phase of implementation focused on piloting a literature review toolkit and testing strategies for bringing the synthesized evidence into decision making processes. Thus the timing of the study did not permit any analysis of: the effectiveness of the new EIDM methods and tools; the extent to which results of literature reviews actually inform or drive decisions; or how evidence informed changes to programs or policies impact public health outcomes or organization operations.

Preliminary themes and insights arising from the analysis were continually fed back to the organization's leaders and staff to allow for member checking of the data and interpretations [41], and to ensure the research contributed real-time value to the unfolding initiative [25,42].

## Results

In this section we briefly describe the presence of EIDM and the EIDM-related activities and changes that occurred in the organization over the first two years of the initiative. The remainder of the results focuses on seven major themes arising from the case study data that address critical factors and dynamics for building capacity to make evidence informed decisions: (1) leadership, (2) organizational structure, (3) human resources, (4) organizational culture, (5) knowledge management, (6) communication, and (7) change management.

### EIDM presence, activities and change: 2008-2010

Prior to fall 2008, before the new Strategic Plan and efforts to advance the EIDM priority, staff were accessing and using research evidence to inform decisions. However, document and informant data showed that across the organization, EIDM-related skills, practices, roles, expectations, resources and products were, for the most part, unclear, inconsistent, individualized, unsystematic, unstructured, implicit, incomplete and/or underdeveloped. Up to this point there were no organization-wide, formalized and standardized methods, tools or expectations to guide and manage how research evidence was brought into decision making processes. Informants believed staff were doing the best job they could with the resources, skills and time available to support this work but they also agreed much more should and could be done to enhance individual and organizational capacity for EIDM.

**Figure 1:**
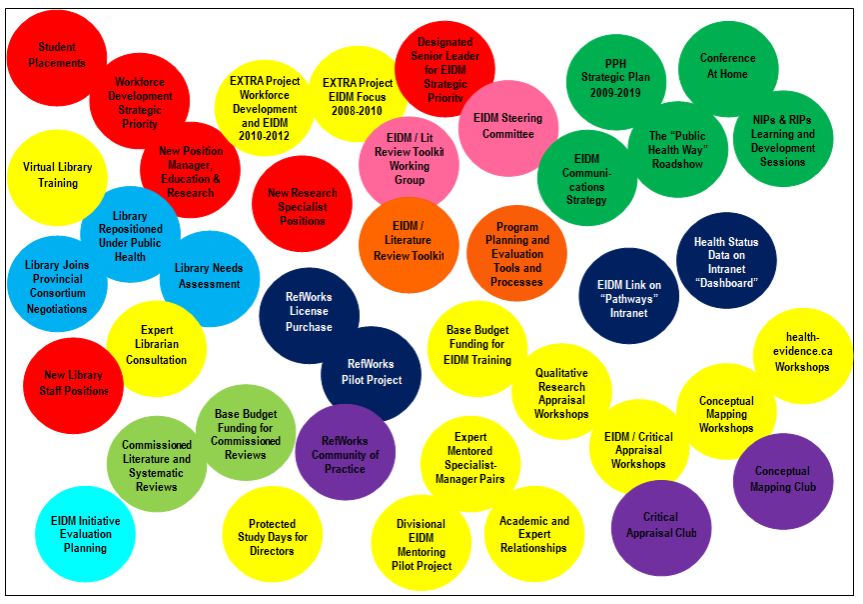
**New EIDM-related activities at PPH, 2008-2010**. Color and position of circles depicts general groups of activities: red--workforce development and staffing; yellow--training and mentoring; dark green--communications; light blue--library restructuring; pink--working groups; orange--toolkits; dark blue--knowledge management; purple--clubs; light green--commissioned reviews; aqua--evaluation.

During the course of this research the organization invested in and advanced significant efforts to nurture a culture and develop the tools, processes and structures that would support, sustain and increase EIDM. These efforts included: offering training and skills enhancement workshops; developing/selecting methods and tools for conducting literature reviews; creating clubs and other forums for sharing knowledge; restructuring the library and expanding its service capacity; creating and supplementing EIDM-related positions; accessing external expertise; commissioning literature reviews; and committing significant base budget funding to EIDM. Figure 1 offers visual depiction of the types, intensity and complexity of new activities undertaken between 2008 and 2010. The colours and positions of the circles represent general groupings of activities. Some activities were initiated and completed in the first two years (e.g., library needs assessment, creation of new staff positions) while many others were ongoing (e.g., clubs, training workshops, literature review toolkit and RefWorks pilot testing) or in development (e.g., evaluation planning, communications plan).

In 2010 staff continued to struggle with key barriers including inadequate access to databases and full-text articles, limited EIDM knowledge and skills, and difficulties allocating sufficient time to engage in EIDM tasks. However, the presence of research evidence and the application of EIDM approaches were more visible and prevalent than in 2008. For example, a literature review toolkit had been developed and was being piloted, EIDM-related skills, practices, roles and expectations were becoming clearer, and in conjunction with the Strategic Plan's Workforce Development priority, a comprehensive training platform for EIDM was in development. Through these training opportunities and experiences with the initiative, staff were gaining confidence and skills and were starting to apply EIDM knowledge, techniques and tools to literature reviews and decision making processes. Unlike the 2008 sample, most divisional/program planning documents and job postings submitted in 2010 contained explicit statements and/or expectations about using EIDM approaches. Newer literature reviews (conducted prior to toolkit piloting) were still inconsistent and incomplete in terms of using and reporting EIDM components (e.g., search strategies, critical appraisal) but compared to earlier reviews these products showed increasing rigor and comprehensiveness. Furthermore, a number of documents revealed significant investments, concerted efforts and widespread activities intended to create or expand fiscal, human, technological and relational resources to advance the EIDM priority.

### Critical factors and dynamics for building EIDM capacity

#### Leadership

Informants agreed that successful implementation and sustainability of the EIDM initiative depended on leadership at the highest level of the organization. One person said "*If the MOH Office is not driving it, it is lost. If they're not onboard, it's not going to happen*."

Across informant groups, the new MOH was seen as a strong, credible and visible leader who provided unwavering support. As a champion of EIDM he has ensured significant and stable funding and staff resources are allocated to pursue the EIDM objectives. Staff thought without the MOH's vision and commitment, this initiative would not have started and they believed his continued involvement will be a key factor contributing to its success.

Another widely recognized leader and champion was the Associate Medical Officer of Health (AMOH) responsible for overseeing implementation of the EIDM strategic priority. Informants thought it was important that a significant portion of this person's time (0.5 FTE) was specifically allocated to EIDM because this provided dedicated resources to move the objectives forward. To enhance leadership capacity and content expertise for this role, the AMOH participated in a two-year mentored executive training program [43]. As part of this fellowship the AMOH carried out a focused intervention project to develop a comprehensive suite of methods and tools that could be used by Specialists and Managers across the organization to conduct consistent, critical and rapid reviews of the research literature.

Informants identified other high level leaders who supported the EIDM initiative including the Chief Administrative Officer and the Commissioner of Health Services for the Region, and the other AMOHs. In 2008, informants were not as certain that all other senior leaders had bought in. However, by winter 2010, with over a year and half of exposure and experience, informants reported more Directors and Managers were demonstrating leadership, commitment and support for the initiative.

The continuity and stability of high level leadership was identified as a key facilitative factor for sustainability of the EIDM initiative. A number of informants predicted that if the current MOH remained and other management positions also stayed fairly stable then the initiative would progress successfully. But if the MOH and/or other key leaders were to leave in the near future, informants questioned whether their replacement(s) would be as committed to this strategic priority. Informants were optimistic that EIDM would eventually spread and take hold, but in 2010 they did not think it was embedded enough in the structures, functions and culture of the organization to withstand a change in key leaders. "*If a new MOH or a new AMOH came in and said 'we're not going to do this,' people wouldn't rally up and say 'you can't take that from us, that's ours and we own that.' It's not there yet*."

#### Organizational structure

In 2008 there was general agreement that the staff and content areas of different divisions and some teams within divisions were not well integrated. Informants suggested more events (e.g., journal clubs, lunch-and-learns) be organized to facilitate ongoing inter-divisional and intra- and inter-role communication, coordination and collaboration. The EIDM initiative created the need and opportunities to build these formal and informal networks. For example, three new clubs were started, each focusing on a different EIDM-related activity: the Critical Appraisal Club, the Conceptual Mapping Club and the RefWorks (reference management software) Community of Practice. These formally organized groups meet regularly to provide opportunities for staff with common interests and responsibilities to develop and practice skills and to discuss challenges and strategies. In 2010 informants reported there had been many more occasions to think, exchange, train and work with colleagues throughout the organization. One person reflected "*I've met a lot of people from other divisions and teams through this EIDM initiative, the club, attending workshops together, getting each other's advice*." Another person simply stated "*Our internal webs are getting tighter*."

One structure that has undergone significant change is the Library. As the initiative progressed it became clear that library services are a vital resource supporting the conduct of effective and efficient literature reviews. One informant explained "*The library has become part of public health. It is structurally, symbolically, and practically starting to have much more of a relationship with the work that we do*." In 2008 not everyone knew about or valued what the Library had to offer. Efforts have since focused on re-orienting the Library (shifting from housing a collection of journals to offering a range of information and reference services) and building the Library's connective and service capacity (e.g., establishing a provincial consortium to improve access to databases and full-text articles, hiring a second full-time librarian, including librarians in EIDM training opportunities). Informants believe these changes are essential to ensure the Library has sufficient capacity, expertise and connections to accommodate the increased demands that are expected with more extensive use of research evidence and more staff using a broader range of library services. As one person expressed "*It's not the physical library that matters. We still have to have some archival material, but what is the library are the people and the interaction and the service*."

#### Human resources

As an organization-wide initiative EIDM is intended for all staff, regardless of their role. Nevertheless, because of functional differences there are some staff groups that will be more exposed to the initiative and more reliant on its approaches, tools and other resources. One of these groups is the Specialists who conduct the literature reviews that are used to inform decision-making. In fall 2008 all but one division had at least two Specialists; by 2010 all divisions had staff in these roles. However, informants agreed that as EIDM becomes routine practice there will be a need to add more of these positions across the organization.

In 2008 senior leaders envisioned a new position that would focus on the Strategic Plan's Workforce Development priority and the Library, both of which intersect with EIDM. In June 2009, PPH hired its first Manager of Education and Research. In the following year the new Manager initiated a staff training survey, coordinated requests for literature reviews, managed the Library restructuring process, and began implementing an organization-wide referencing system. Moving forward, one of this Manager's main responsibilities is to plan and coordinate a comprehensive, organization-wide training platform for EIDM.

Framing researchable questions, designing and conducting efficient and effective searches and critically appraising research evidence requires well-developed knowledge and high-level skills. In 2008 very few staff had expertise in these areas and limited training was available. However, between 2008 and 2010 dozens of staff attended half- or one-day introductory EIDM sessions, intensive week-long EIDM workshops, and/or two-day workshops on qualitative research appraisal. In addition, internal and external EIDM experts began coaching Specialist-Manager pairs through systematic reviews of the research literature to answer specific practice-based questions. Overall, informants thought the new training opportunities had strengthened and expanded not only their knowledge and skills, but also their internal and external relationships. One person shared "*I think it was just as much an EIDM adventure as it was a team building exercise*." Someone else said "*I appreciated the opportunity to go to the qualitative workshop. That was amazing*." Another informant added "*My network of people outside the organization who can help has grown exponentially*."

Clearly staff were being trained, but in the first two years there was not enough money, opportunity or time to train everyone. Informants agreed it was appropriate to launch the initiative with a focus on the Specialists and Managers, but in 2010 many thought it was time to shift some attention and resources into training others. One person asserted "*People are saying, when will it be my turn? If we say wait, it's coming, people will get frustrated. We have to put something on the table*." In the meantime informants said those who get training need to share what they learn and the resources they acquire with colleagues. Team and divisional leaders thought guidelines need to be developed to inform decisions about which staff to send for what types and levels of training and when. As one person remarked "*Things aren't zero or one with EIDM. It's not like there is only one package. It's understanding what intensity of training folks need to be effective contributors to the process*."

Some informants talked about annual reviews as a means of providing regular and mutually accountable opportunities for staff and supervisors to identify EIDM learning needs, develop training plans, monitor progress, and assess performance. They said if the organization is promoting EIDM as a strategic priority and expects EIDM as part of routine practice then it should be explicitly added to the review and appraisal processes and products. Although not included in the formal templates, in 2010 several informants reported they had incorporated EIDM in their performance objectives and they would be using their annual reviews to demonstrate how they are developing and applying EIDM knowledge and skills.

#### Organizational culture

Informants agreed there must be a fundamental shift in the culture of the organization to support EIDM. One person explained "*You can't just walk in and say, you're going to do it like this, and suddenly it happens. You've got to change the culture first and get people believing before they'll actually do it*." When asked to describe what this would look like, informants talked about a multi-faceted culture of: inquiry, curiosity, critical consumerism, supported risk taking, constructive challenge, quality improvement, excellence, innovation, trust, respect, reflection, reasoning, learning and sharing. In 2008 informants had a clear sense that the organization was ready to pursue this change. Some individuals were anxious, but for the most part staff were curious and interested in seeing the initiative move forward. In 2010 organizational readiness for EIDM-related changes continued to be strong and informants reported a positive increase in overall staff acceptance, support and commitment.

A commonly identified obstacle to embracing and integrating EIDM was the lack of time to read, to think, to write. In 2008 and 2010 informants agreed the organization reflected a culture of "*doing*." Many informants said staff are often overwhelmed with the demands of practice, have difficulties giving up tasks, and don't feel empowered to say no. There was a sense that enabling staff to make and take the time for EIDM will require more balance between thinking and doing. However, informants thought staff would struggle with the shift to a "*thinker-doer*" culture without on-going encouragement and explicit permission from senior leaders to designate and protect sufficient time to engage in the steps of EIDM. "*We don't want to go from a culture of doing, doing, doing, to a culture of people feeling frustrated that they're not getting that done AND they're not doing enough thinking*."

#### Knowledge management

In 2008 the health department did not have an organization-wide system to manage all of the information used and produced by literature reviews and decision making processes. As one person remarked "*There are piles of papers all over this place*." Some divisions/teams had created databases or hard-copy files to store documentation, but these dossiers were limited in content and functionality. In 2010 informants emphasized the need to develop a comprehensive knowledge management strategy that would provide a platform for coherent and transparent EIDM processes, full and rapid acquisition of information, consistent and thorough documentation, and the ability to share knowledge across the organization.

When asked to describe the essential features of a knowledge management system informants said it should: be easily accessible, user-friendly, current, electronic and searchable; include all EIDM tools, templates and manuals; link directly to companion resources (e.g., Library services, reference management software); contain an "evidence repository" that would include completed search strategies/results, critical appraisal forms and literature reviews; and have the capacity to generate a comprehensive audit trail for every decision. The organization's intranet site was identified as a logical and familiar location for the knowledge management system. However, informants cautioned against an "if you build it they will come" mentality. They said it will be important to build awareness and provide prompts and training so staff want to, and know how to, navigate and use the system. "*Just because things are on [the intranet] doesn't mean people are going to use them. There needs to be some thought around how we entice staff to open and use that link*." In 2010 plans were underway to develop and populate the evidence repository with completed literature reviews.

#### Communications

In 2008 participants were asked what they had heard about EIDM and the organization's EIDM initiative. People remembered the MOH introducing the concept at an All-Staff Day in 2007 and appearances by senior leaders at divisional meetings in 2008 to familiarize staff with the forthcoming Strategic Plan and the EIDM priority. Most informants had at least heard the terms "evidence informed decision making" and "EIDM." However it would take more than these initial presentations to effectively reach and inform all staff. Informants expected and wanted more communication and more clarity about EIDM and how the impending changes would influence individual and organizational practices.

When asked the same question in 2010, participants talked about EIDM as a key component of the Strategic Plan which was formally released in 2009. Many informants also mentioned that EIDM was the main theme of PPH's first intra-organizational conference in 2009. Some staff had heard more about EIDM and the initiative through their involvement on the literature review toolkit working group, in training sessions and/or during senior management committee discussions. The document and informant data showed the language of EIDM had started to permeate the organization. "*Before EIDM nobody knew what the acronym stood for and now more people across the board know what that stands for*." "*Staff are more comfortable using the terminology of EIDM. It's in their minds, in their conversations*." While exigent circumstances were acknowledged (i.e., the H1N1 outbreak), informants also agreed that not enough had been done in the first two years to spread the EIDM message across the entire organization and that a key priority moving forward would be to implement a comprehensive organization-wide communications strategy for EIDM.

#### Change management

Informants thought explicitly framing EIDM capacity building as a long-term change process allows sufficient time to set, pursue and achieve realistic individual and organizational goals. One person explained "*That's why we chose that 10 year horizon. These are truly strategic, big things. If you had even a 3 year Strategic Plan, at the end of it you would say, well that was a waste of time, didn't get anything useful done, and then you give up. This way you say, it's a huge job but we can see we're making progress*."

In 2008, expectations and actions focused on selecting/developing methods and tools for conducting literature reviews and training for Specialists and Managers. By 2010 it was obvious EIDM-related changes had been and would be much broader and more extensive. According to informants, designing and implementing the initiative has been an evolutionary and organic process. Activities and training options were added and adapted to respond to emerging and anticipated needs, challenges and opportunities. While recognizing progress, in 2010 some informants commented on the need for more comprehensive management of the EIDM initiative and co-management of EIDM and other Strategic Plan priorities (e.g., Workforce Development, Performance Management) and other organizational/divisional initiatives. As illustrated in Figure 1 many appropriate and intentional EIDM-related activities were undertaken in the first two years but they were not strategically connected and/or sequenced. One person reflected, "*Change management may not have been as salient in those early conversations and actions, whereas it has grown into something that is requiring more strategy. It's more complex than people first imagined*."

While the mechanics of EIDM remained a priority, by 2010 the humanistic side of the initiative was drawing attention. One person said "*It's basically getting people to think about different ways of doing things. There's a whole set of feelings around what that means, so it's not enough to put all our energies into the evidence piece*." Another person used the analogy of riding a roller coaster to describe the emotional impacts. "*There is tension between, this is kind of exciting, and I'm not sure that I'm comfortable with this. The challenge is in the balance of that. We want staff to get off the [EIDM] ride and say, 'Wow, I'd like to go on again!*'" Initially, emotional responses were tied to anxieties related to learning new skills (e.g., critical appraisal) or mastering advanced skills. These concerns were dissipating as staff received training and had opportunities to practice skills. In 2010 the emotional issues were shifting to potential impacts of decisions. At the outset of the initiative it was expected that rigorous evidence reviews informing decisions would lead to practice changes. But until 2010 the consequences of EIDM had not been felt. The impact was just becoming real for staff as the first reviews using the new methods and tools were being completed and used to inform decision making. Informants raised concerns about tensions that may arise, internal to the organization and with external groups, if literature review results and/or decisions challenge the status quo. Managers appealed for training and other supports to equip themselves and their staff to handle such situations, confidently and successfully.

## Discussion

Political visions, practice standards, knowledge and skills, and critical appraisal tools are necessary but not sufficient to ensure effective and efficient EIDM. The characteristics and capacity of public health organizations are also key [1,44-48]. As discussed below, the facilitators and barriers of organizational change identified in this research are similar to themes highlighted by others who have studied the "active ingredients" [49] of system transformations to promote EIDM and/or quality improvement in public health and other health-related sectors.

System forces, such as national practice standards may compel EIDM-related reforms, but leaders of health care organizations are the "endogenous catalysts" [50] that stimulate and propel on-the-ground change [1,49-55]. Research has identified attributes and behaviours of effective leaders of organizational transformation for EIDM, including setting, steering and staying the course for change, becoming active participants in change efforts [53,56], the readiness and ability to secure and (re)allocate human, material and fiscal resources, and nurturing a culture that is open to change and values the inclusion of research evidence in decision making [49,54]. One aspect of leadership the literature does not emphasize that was identified in this study is stability. Long-term involvement of a consistent group of senior leaders has reinforced the presence, prominence and permanence of PPH's EIDM initiative.

Consistent with other research on organizational change and capacity building for EIDM [50,51,53,55,56], the findings of this study demonstrate the value of enhancing formal and informal relational structures. Clubs, committees and other groups provide opportunities for staff to get involved, exchange ideas, gain experience, assume responsibility, and take ownership. Structures that bring staff together are important, but some authors argue the pressing priority for advancing EIDM is building organizational structures that facilitate access to knowledge [1,50,52,54]. Strategic goals, critical appraisal skills and enthusiasm for EIDM are of limited use if organizations lack the infrastructure to acquire research evidence. It is not enough for public health professionals to rely on academic or personal connections to help find and obtain research for decision making. These organizations need direct and easy access to technology for EIDM, to information specialists and to full-text research literature. Recognizing the critical importance of this aspect of capacity building for EIDM, PPH has invested significant time, effort and funds to develop its internal library infrastructure and expertise. Understanding that similar efforts and investments may be difficult for smaller, remote and/or less well funded health departments, there is a need for organizations (both those endowed and those in need) as well as the provincial and national public health systems, to explore opportunities for staff and IT resource sharing, using virtual networks, creating consortiums to procure group rights to access databases and journals of restricted circulation, and encouraging publication of relevant research in open access sources.

There is agreement in the literature and with the findings of this study that, in general, the public health workforce lacks research methods and critical appraisal skills, and that more formal and advanced training is needed on the concepts, tools, technologies and applications of EIDM [49,50,52]. At the outset of PPH's initiative, very few staff had the requisite skills to conduct efficient and effective reviews of the literature but by 2010 a large number of staff had participated in EIDM training workshops and plans were underway for an organization-wide training platform. By designating a significant portion of a senior leader's time to advance the EIDM priority and creating several new positions dedicated to EIDM-related work, PPH also counteracted concerns about negative impacts on performance when efforts are under-staffed or rely on volunteers [1,50]. Including EIDM-related expectations and opportunities within an organization's performance, accountability and incentive structures is another facilitative factor identified in the literature [49,51-53,55,57] and in the findings of this study. What the literature does not address but was critical to advance training and increase the number of EIDM-related staff positions in this case, was the organization's decision to commit significant long-term core funding for these activities and salaries.

A supportive culture has been identified as a key contextual determinant of change to promote EIDM in health-related settings [55]. The results of this study mirror what other authors [49,52,53,55] suggest are key characteristics of such cultures, for example: valuing people, learning, and the use of research evidence; encouraging innovation, out-of-the-box thinking and risk-taking; and making time for critical reflection a priority. Compared to selecting tools, training staff and other more technical and/or discrete aspects of EIDM capacity building, changing the culture of an organization is a much harder and longer process [58]. As demonstrated in this study, there is value in beginning with a long-term Strategic Plan that explicitly anticipates and allows sufficient time for EIDM to become part of the everyday and expected routines of the organization and its workforce.

There is increasing recognition of the critical importance of knowledge management for effective EIDM [1,59]. As learned in this study, EIDM approaches require, use and produce significant volumes of information. However, the structures, technology and expertise within organizations either do not exist or are not are not well matched to manage this knowledge. Capacity building efforts at PPH included plans to create an in-house knowledge management system. While there is certainly value in an organization taking steps to manage its internal knowledge, there would be greater value in a comprehensive knowledge management system that serves the public health sector as a whole. The application of knowledge may be different across settings, but the issues facing health units and the sources of research on these problems would likely be the same or very similar. Making available the work already done by one organization to synthesize, appraise and use evidence for decision making to others would contribute to maximizing efficiencies, reducing duplication, and increasing transparency and consistency. To this end, some internet-based platforms have been developed to help improve access to, and retrieval and use of scientific evidence and other forms of knowledge for decision making in public health [e.g., 60]. In addition, public health leaders in Canada have begun thinking and talking about developing a national strategy for knowledge management [61,62]; operationalizing this vision, though challenging, would provide much needed system level capacity for EIDM.

A number of authors address the importance of and mechanisms for communication in organizational change and capacity building for EIDM. They emphasize the need for senior leaders to communicate early and continuously about the rationale, plans, activities, progress and practical implications of change [54,57]. They also discuss designating and using multiple channels to increase awareness, promote dialogue, and generate widespread buy-in and adoption [1,54,56]. Furthermore, research has demonstrated the value of developing a comprehensive communication strategy that includes dedicated resources [50]. While the nature of communication regarding the EIDM initiative reflected some of the qualities described in the literature, this is an area where more concerted and systematic efforts are needed at PPH. Informants in this study recognized the lack of and need for an organization-wide, comprehensive, EIDM-specific communication plan and senior leaders indicated that developing and resourcing this strategy would be a key priority of future efforts.

Crow [51] states "we are beginning to realize that the change itself is not usually the problem. The problem is our reaction to change" (p. 239). Like Crow, many authors [52,53,57,63] identify the need for management strategies that help leaders acknowledge and address staff emotions related to change, new expectations and altered responsibilities. Efforts to build capacity for EIDM must focus on the tasks and resources required to conduct evidence reviews, but to be successful, they must also identify and respond to the needs of the people who perform this work [57]. The findings of this study reinforce the utility of change management frameworks and highlight the importance of recognizing and addressing the range of negative and positive emotional reactions to EIDM and to organizational change.

It is clear there are many catalysts and components of organizational change to promote EIDM. Implementing a comprehensive EIDM strategy is similar to the implementation of large scale enterprise technologies such as customer relationship management (CRM) or performance measurement systems. It requires contextual preparation, incremental efforts, adaptive capacity, on-going resource investments, attention to human needs, and an awareness of the interdependence of intervention elements and stakeholder groups. This case study demonstrates the complexity and expansiveness of the activities, factors and dynamics involved in making EIDM a standard feature of public health practice. It also reinforces the notion that EIDM cannot be pursued or achieved by an organization in isolation. Partnerships with other health settings, access to external knowledge sources, and inputs from provincial and national public health agencies are necessary to realize EIDM's potential to have consequential and sustainable impacts on public health services and health outcomes.

### Limitations

There are several limitations of this research. First, as a single-site case study no assumptions can be made regarding the generalizability of the findings. Public health and other health care organizations interested in applying the knowledge will need to consider contextual similarities and differences to assess the theoretical transferability of the findings to their unique settings [41]. Second, the scope of the study focused only on the first two years of a 10-year strategic initiative. Thus, while the findings capture a critical period of organizational change and implementation, they cannot anticipate the future progression, facilitators and/or barriers of efforts to build EIDM capacity. The timeframe of the study also precluded any evaluation of actual performance or outcomes related to EIDM. Finally, the decision was made to only include staff responsible for literature reviews and individuals most involved in the initial roll out of the EIDM initiative. Therefore, this study does not consider the perspectives of the front-line professionals who will eventually be involved in and impacted by the organization-level change.

### Future research

Further research is needed to expand our understanding of, and provide practical guidance for organizational capacity building for EIDM. It will be important to continue studying the characteristics and effectiveness of strategies used to increase and improve uptake of research evidence in health care decision making. Individual organizations planning or undergoing changes to promote EIDM would benefit from developmental and formative evaluations [28,42] that can offer practical, critical and real-time insights and assessments to inform and/or re-calibrate efforts. Studies that include after action reviews [64] and summative evaluations [31] are needed to demonstrate if and how EIDM approaches and organizational changes are actually impacting public health policy and practice. Longitudinal research will be important to assess the sustainability of organizational changes and strategies to promote EIDM. It would be useful if research could identify the appropriate combination, sequence, duration, intensity and audiences for the range of EIDM-related activities. To contribute to a more general theory of organizational capacity building for EIDM, multi-site case studies are needed that compare and contrast the dynamics, resources, mechanisms and impacts of EIDM initiatives in different organizational contexts. Finally, public health is not the only sector pursuing efforts to build organizational capacity for EIDM [e.g., 55,65-67] and there is much that could be learned and achieved through cross-disciplinary collaborations.

## Conclusion

As demonstrated in this study, the process of building capacity to become an evidence informed decision making organization involves a number of key features and dynamics. It needs a vision and strong leadership to move it forward. It involves many different staff and it positions staff in new working relationships. It expects proficiencies in EIDM skills and necessitates the provision of training opportunities. It requires significant fiscal and technological resources. It induces a shift in the culture of the organization from "doer" to "thinker-doer." It demands a knowledge management strategy to ensure evidence and decision making information is current, comprehensive, accessible, usable and evaluable. It necessitates a communications strategy to raise awareness, develop shared vocabulary, provide updates, and maintain clarity and transparency. And, it compels careful monitoring, management, and evaluation of the mechanistic and humanistic aspects and outcomes of change. With leadership, planning, commitment and substantial investments, in the first two years of a 10-year initiative, a public health department made significant progress towards achieving its goal of becoming an evidence informed decision making organization.

## Abbreviations

AMOH: Associate Medical Officer of Health; EIDM: Evidence Informed Decision Making; MOH: Medical Officer of Health; PPH: Peel Public Health.

## Competing interests

The authors declare that they have no competing interests.

## Authors' contributions

All authors conceived the study. LP designed the study, conducted the interviews, collected the documents, analyzed the data, and drafted the manuscript. DC and MD provided assistance with data analysis. DC, MD and DM participated in the design of the study, were available for consultation throughout the study, offered critical input and revisions on drafts of the manuscript, and approved the final version.

## Pre-publication history

The pre-publication history for this paper can be accessed here:

http://www.biomedcentral.com/1471-2458/12/137/prepub

## Supplementary Material

Additional file 1 **Interview/focus group guide**. Click here for file

## References

[B1] Bowen S Erickson T Martens P Crockett S More than "using research": the real challenges in promoting evidence-informed decision-making Healthcare Policy 2009 4 87 102 19377360 PMC2653695

[B2] Campbell JA The SARS Commission interim report--SARS and public health in Ontario 2004 Ontario Ministry of Health and Long-Term Care http://www.health.gov.on.ca/english/public/pub/ministry_reports/campbell04/campbell04.html 10.1089/153871304323146423 15225406

[B3] Dobbins M DeCorby K Twiddy T A knowledge transfer strategy for public health decision makers Worldviews Evid Based Nurs 2004 1 120 128 10.1111/j.1741-6787.2004.t01-1-04009.x 17129325

[B4] Dobbins M Hanna SE Ciliska D Manske S Cameron R Mercer SL O'Mara L DeCorby K Robeson P A randomized controlled trial evaluating the impact of knowledge translation and exchange strategies Implement Sci 2009 4 61 10.1186/1748-5908-4-61 19775439 PMC2936828

[B5] Kirby MJL LeBreton M Reforming health protection and promotion in Canada: time to act 2003 Ottawa, ON: The Standing Senate Committee on Social Affairs, Science and Technology http://www.parl.gc.ca/37/2/parlbus/commbus/senate/com-e/soci-e/rep-e/repfinnov03-e.pdf 15230165

[B6] Lapelle N Luckmann R Simpson EH Marin E Identifying strategies to improve access to credible and relevant information for public health professionals: a qualitative study BMC Publ Health 2006 6 89 10.1186/1471-2458-6-89 PMC1456961 16597331

[B7] Naylor D Basrur S Bergeron MG Brunham RC Butler-Jones D Dafoe G Ferguson-Paré M Lussing F McGeer A Neufeld KR Plummer F Learning from SARS: renewal of public health in Canada. (Report No 1210) 2003 Ottawa, ON: Health Canada http://www.phac-aspc.gc.ca/publicat/sars-sras/naylor/

[B8] Brownson RC Gurney JG Land GH Evidence-based decision making in public health J Public Health Manag Pract 1999 5 86 97 10558389 10.1097/00124784-199909000-00012

[B9] Ciliska D Introduction to evidence informed decision making. On-line learning module 2009 Ottawa, ON: Canadian Institutes of Health Research http://www.learning.cihr-irsc.gc.ca/

[B10] Kohatsu ND Robinson JG Torner JC Evidence-based public health: an evolving concept Am J Prev Med 2004 27 417 421 15556743 10.1016/j.amepre.2004.07.019

[B11] National Collaborating Centre for Methods and Tools A model for evidence informed decision making in public health 2009 Hamilton, ON http://www.nccmt.ca/pubs/FactSheet_EIDM_EN_WeB.pdf 10.14745/ccdr.v47i56a08 PMC8219060 34220355

[B12] Medlar B Mowat D Di Ruggiero E Frank J Introducing the national collaborating centres for public health Can Med Assoc J 2006 175 493 494 10.1503/cmaj.060850 16940269 PMC1550741

[B13] Public Health Agency of Canada Core competency statements 2008 Ottawa, ON http://www.phac-aspc.gc.ca/ccph-cesp/stmts-enon-eng.php

[B14] Ontario Ministry of Health and Long Term Care Ontario public health standards 2008 2008 Toronto, ON http://www.health.gov.on.ca/english/providers/program/pubhealth/oph_standards/ophs/progstds/pdfs/ophs_2008.pdf

[B15] Canadian Centre for Evidence-Based Nursing Evidence informed decision making (EIDM) workshop 2010 Hamilton, ON http://ccebn.mcmaster.ca/

[B16] National Collaborating Centre for Methods and Tools On-line learning module: introduction to evidence informed decision making 2010 Hamilton, ON http://www.nccmt.ca/en/modules/eidm/

[B17] Public Health Agency of Canada Skills enhancement for public health 2007 Ottawa, ON http://www.phac-aspc.gc.ca/sehs-acss/training_modules-eng.php

[B18] Alberta Health Services Evidence-based Practice Centre Putting evidence into practice (PEP) workshop 2011 Edmonton, AB http://www.pep.ualberta.ca/

[B19] Kothari A Birch S Charles C "Interaction" and research utilisation in health policies and programs: does it work? Health Policy 2005 71 117 125 10.1016/j.healthpol.2004.03.010 15563998

[B20] Jewell CJ Bero LA "Developing good taste in evidence": facilitators of and hindrances to evidence-informed health policymaking in state government Milbank Q 2008 86 177 208 10.1111/j.1468-0009.2008.00519.x 18522611 PMC2690362

[B21] Scott C Seidel J Bowen S Gall N Integrated health systems and integrated knowledge: creating space for putting knowledge into action Healthc Q 2009 13 30 36 20057246 10.12927/hcq.2009.21094

[B22] Canadian Health Services Research Foundation How an RHA organized itself to better integrate evidence into decision-making Promising Practices in Research Use 2007 Ottawa, ON http://www.chsrf.ca/publicationsandresources/pastseries/PromisingPracticesinResearchUse/article/07-01-01/3629bf20-e641-45bd-8974-f8dc2560d6ef.aspx

[B23] Canadian Health Services Research Foundation Bringing knowledge home: embedding evidence in decision-making Promising Practices in Research Use 2006 Ottawa, ON http://www.chsrf.ca/publicationsandresources/pastseries/PromisingPracticesinResearchUse/article/06-05-01/3a723264-4829-409b-8f5d-2d33fd232742.aspx

[B24] Canadian Health Services Research Foundation Buying time and getting a bonus: how a regional health organization is tackling a barrier to building research use capacity and discovering additional benefits Promising Practices in Research Use 2008 Ottawa, ON http://www.chsrf.ca/publicationsandresources/pastseries/PromisingPracticesinResearchUse/article/08-02-01/60a26d7c-edc4-40a0-8e79-fecb1c61a5de.aspx

[B25] Graham ID Logan J Harrison MB Straus SE Tetroe J Caswell W Robinson N Lost in knowledge translation: time for a map? J Contin Educ Health Prof 2006 26 13 24 10.1002/chp.47 16557505

[B26] Dobbins M Kyle R Timmings C Ward M Cava M Clarke C Pietrusiak M Akhtar-Danesh N Jack S Kothari A Lemieux-Charles L McKibbon A Peirson L Sibbald S A Tailored, Collaborative Strategy to Develop Capacity and Facilitate Evidence-Informed Public Health Decision Making Hamilton: Funding Agency: Canadian Institutes of Health Research 2009-2012

[B27] Peel Public Health 2009-2019--Staying ahead of the curve: Peel Public Health's 10-year strategic plan 2009 Brampton, ON http://www.peelregion.ca/health/health-status-report/stay-ahead-curve/

[B28] Patton MQ Qualitative research and evaluation methods 2002 3 Thousand Oaks, CA: Sage

[B29] Stake RE Denzin NK, Lincoln YS Qualitative case studies Handbook of qualitative research 2005 3 Thousand Oaks, CA: Sage 443 466

[B30] Yin RK Case study research: design and methods 2009 4 Los Angeles: Sage

[B31] Denzin N The research act: a theoretical introduction to sociological methods 1989 3 Englewood Cliffs, NJ: Prentice-Hall

[B32] National Collaborating Centre for Methods and Tools Stages in the evidence-informed public health process Hamilton, ON http://www.nccmt.ca/eiph/index-eng.html

[B33] Rogers EM Diffusion of innovations 2003 5 New York: Free Press

[B34] Senge PM The fifth discipline: the art & practice of the learning organization 2006 New York: Doubleday

[B35] Greenhalgh T Robert G Macfarlane F Bate P Kyriakidou O Diffusion of innovations in service organizations: systematic review and recommendations Milbank Q 2004 82 581 629 10.1111/j.0887-378X.2004.00325.x 15595944 PMC2690184

[B36] Kotter JP Leading change 1996 Boston, MA: Harvard Business Press

[B37] Cawsey T Deszca G Toolkit for organizational change 2007 Los Angeles: Sage

[B38] Social Care Institute for Excellence Learning organizations: a self-assessment resource pack 2008 London, UK http://www.scie.org.uk/publications/learningorgs/index.asp

[B39] Stetler CB Updating the Stetler model of research utilization to facilitate evidence-based practice Nurs Outlook 2001 49 272 279 10.1067/mno.2001.120517 11753294

[B40] Rycroft-Malone J The PARIHS framework--a framework for guiding the implementation of evidence-based practice J Nurs Care Qual 2004 19 297 304 10.1097/00001786-200410000-00002 15535533

[B41] Lincoln YS Guba EG Naturalistic inquiry 1985 Newbury Park, CA: Sage

[B42] Patton MQ Developmental evaluation: applying complexity concepts to enhance innovation and use 2010 New York: The Guilford Press

[B43] Canadian Health Services Research Foundation About EXTRA Ottawa, ON http://www.chsrf.ca/extra/about_e.php

[B44] Kitson A Ahmed LB Harvey G Seers K Thompson DR From research to practice: one organizational model for promoting research-based practice J Adv Nurs 1996 23 430 440 10.1111/j.1365-2648.1996.tb00003.x 8655816

[B45] Battista RN Innovation and diffusion of health-related technologies: a conceptual framework Int J Technol Assess Health Care 1989 5 227 248 10.1017/S0266462300006450 10303488

[B46] McCaughan D Thompson C Cullum N Sheldon TA Thompson DR Acute care nurses' perceptions of barriers to using research information in clinical decision-making J Adv Nurs 2002 39 46 60 10.1046/j.1365-2648.2002.02241.x 12074751

[B47] Forsetlund L Bjorndal A Identifying barriers to the use of research faced by public health physicians in Norway and developing an intervention to reduce them J Health Serv Res Policy 2002 7 10 18 10.1258/1355819021927629 11822256

[B48] McWilliam CL Kothari A Kloseck M Ward-Griffin C Forbes D Organizational learning for evidence-based practice: a 'PAKT' for success J Chang Manag 2008 8 233 247 10.1080/14697010802397016

[B49] Brownson RC Fielding JE Maylahn CM Evidence-based public health: a fundamental concept for public health practice Ann Rev Public Health 2009 20 175 201 19296775 10.1146/annurev.publhealth.031308.100134

[B50] Hamel N Shrecker T Unpacking capacity to utilize research: a tale of the Burkina Faso public health association Soc Sci Med 2011 72 31 38 10.1016/j.socscimed.2010.09.051 21074923

[B51] Crow G Diffusion of innovation: the leaders' role in creating the organizational context for evidence-based practice Nurs Admin Q 2006 30 236 242 10.1097/00006216-200607000-00008 16878009

[B52] Kitson A Harvey G McCormack B Enabling the implementation of evidence based practice: a conceptual framework Qual Health Care 1998 7 149 158 10.1136/qshc.7.3.149 10185141 PMC2483604

[B53] Riley WJ Parsons HM Duffy GL Moran JW Henry B Realizing transformational change through quality improvement in public health J Public Health Management Practice 2010 16 72 78 10.1097/PHH.0b013e3181c2c7e0 20009648

[B54] Newhouse RP Creating infrastructure supportive of evidence-based nursing practice: leadership strategies Worldviews on Evidence-Based Nursing 2007 4 21 29 10.1111/j.1741-6787.2007.00075.x 17355407

[B55] Stetler CB Ritchie JA Rycroft-Malone J Schultz AA Charns MP Institutionalizing evidence-based practice: an organizational case study using a model of strategic change Implement Sci 2009 4 78 10.1186/1748-5908-4-78 19948064 PMC2795741

[B56] Edwards N Grinspun D Understanding whole systems change in healthcare: the case of emerging evidence-informed nursing service delivery models 2011 Ottawa, ON: Canadian Health Services Research Foundation http://www.chsrf.ca/Libraries/OGC_Reports/Edwards-Grinspun-EN.sflb.ashx

[B57] Thompson JM Understanding and managing organizational change: implications for public health management J Public Health Management Practice 2010 16 167 173 10.1097/PHH.0b013e3181c8cb51 20150801

[B58] Potter C Brough R Systemic capacity building: a hierarchy of needs Health Policy Plan 2004 19 336 345 10.1093/heapol/czh038 15310668

[B59] Armstrong R Waters E Roberts H Oliver S Popay J The role and theoretical evolution of knowledge translation and exchange in public health J Public Health 2006 28 384 389 10.1093/pubmed/fdl072 17082462

[B60] Dobbins M DeCorby K Robeson P Husson H Trillis D Greco L A knowledge management tool for public health: health-evidence.ca BMC Public Health 2010 10 496 10.1186/1471-2458-10-496 20718970 PMC2936422

[B61] National Collaborating Centre for Methods and Tools Proceedings of NCCMT's 2008 knowledge management in public health conference 2009 Hamilton, ON http://www.nccmt.ca/tools/publications-eng.html

[B62] Dubois N Wilkerson T Knowledge management strategy forum summary report: a synopsis of the November 5th 2008 discussion 2009 Hamilton, ON: National Collaborating Centre for Methods and Tools http://www.nccmt.ca/tools/publications-eng.html

[B63] Campbell RJ Change management in health care Health Care Manag 2008 27 23 39 10.1097/01.hcm.0000285028.79762.a1 18510142

[B64] Robertson S After Action Reviews 2005 National Institute for Health and Clinical Excellence https://www.evidence.nhs.uk/document?ci=http%3A%2F%2Farms.evidence.nhs.uk%2Fresources%2FHub%2F21142&ReturnUrl=%2Fsearch%3Fq%3Drobertson%2Bafter%2Baction%2Breview

[B65] Aarons GA Hurlburt M McCue Horwitz S Advancing a conceptual model of evidence-based practice implementation in public service sectors Adm Policy Ment Health 2001 38 4 23 21197565 10.1007/s10488-010-0327-7 PMC3025110

[B66] Glisson C Schoenwald S The ARC organizational and community intervention strategy for implementing evidence-based children's mental health treatments Ment Health Serv Res 2005 7 243 259 10.1007/s11020-005-7456-1 16320107

[B67] Jack SM Dobbins M Sword W Novotna G Brooks S Lipman EL Niccols A Evidence-informed decision-making by professionals working in addiction agencies serving women: a descriptive qualitative study Subst Abuse Treat Prev Policy 2011 6 29 10.1186/1747-597X-6-29 22059528 PMC3224771

