# Supplementary material for: Building capacity for evidence informed decision making in public health: a case study of organizational change
Source: BMC Public Health. 2012 Feb 20;12:137. doi: 10.1186/1471-2458-12-137 (PMC3305606; doi:10.1186/1471-2458-12-137)
Supplement: Additional file 1 — Interview/focus group guide. [file 1471-2458-12-137-S1.PDF]

1. How are policy and program decisions normally made in your organization? What are the steps taken when preparing for and engaging in decision making?
2. To what extent is research evidence routinely used to inform program and policy decisions in this organization? How do staff go about finding, accessing, assessing, and applying research evidence to decision making processes?
3. What are some of the personal and/or organizational factors that act as barriers or facilitators for staff to be able to find or locate research evidence?
4. What are some of the personal and/or organizational factors that act as barriers or facilitators for staff to be able to access or get their hands on research evidence?
5. What are some of the personal and/or organizational factors that act as barriers or facilitators for staff to be able to assess the quality of research evidence?
6. What are some of the personal and/or organizational factors that act as barriers or facilitators for staff to be able to apply research evidence to the local context or to use the evidence to meaningfully support local decision making about public health policies and programs?
7. What are the most significant obstacles the organization must overcome if it is going to be successful in enhancing the ability of its staff to find, access, assess, and use research findings to support policy and program decision making? How might these barriers be minimized or eliminated?
8. What are the most important things the organization has or could have going for it to facilitate moving toward its EIDM goals? How might the organization maximize these positive aspects?
